# Cell- and sex-specificity in the transcriptomic response of the hippocampal neurovascular unit to obesity

**DOI:** 10.1038/s42003-025-09112-6

**Published:** 2025-11-27

**Authors:** Jennifer E. Norman, Saivageethi Nuthikattu, Dragan Milenkovic, Amparo C. Villablanca

**Affiliations:** 1 Division of Cardiovascular Medicine, Department of Internal Medicine, University of California, Davis, Davis, CA USA; 2 Department of Nutrition, University of California, Davis, Davis, CA USA; 3 Plants for Human Health Institute, Department of Food, Bioprocessing and Nutrition Sciences, North Carolina State University, Kannapolis, NC USA

**Keywords:** Transcriptomics, Dementia, Obesity

## Abstract

Obesity is a risk factor for vascular dementia and exhibits sex differences in comorbidities and prevalence. However, its cell-specific effects on the neurovascular unit (NVU) remain unknown. Here we show, using the *ob/ob* mouse model and single nuclei RNA sequencing, how obesity modulates the hippocampal NVU transcriptome in females. Obesity alters endothelial-specific differentially expressed genes (DEGs) involved in angiogenesis and blood brain barrier permeability. DEGs in common between endothelial cells and other NVU cells are associated with neurotransmission and autophagy. Some gene expression changes from each NVU cell type correlate with behavioral changes. Sex-based analyses comparing to our previously reported male data indicate that the NVU transcriptomic response to obesity is modified by sex in a cell-type specific manner. Endothelial and microglial cells exhibit greater sex-specificity than astrocytes and neurons. The findings from our work may help inform both sex-independent and sex-specific approaches to prevention and treatment for obesity-related dementia.

## Abstract (web summary)

Single nuclei RNA sequencing of the *ob/ob* mouse hippocampus uncovers cell type- and sex-specific obesity-induced alterations in the neurovascular unit transcriptome, with implications for obesity-associated cognitive decline.

## Introduction

Obesity, a chronic multifactorial disease, is a growing global epidemic affecting more than a billion people worldwide^1,2^. The rise in obesity is associated with a corresponding increase in the risk for its comorbidities such as type 2 diabetes mellitus (T2DM), cardiovascular disease (CVD)^3,4^, and neurodegenerative diseases, including dementias^5^. Growing evidence suggests the vascular effects of obesity play a significant role in the development of vascular dementia^6^ via dysfunction of the cerebral microcirculation^7^. The pathology of vascular dementia involves disruption of the neurovascular unit (NVU)^8,9^ which consists of endothelial cells, microglial cells, astrocytes, and neurons^10^.

Obesity induces alterations in the cerebral vasculature, oxidative stress, blood-brain barrier (BBB) and neuroinflammation, via cerebral hypoperfusion^11,12^. This contributes to endothelial cell dysfunction, dysregulation of neurons and glial cells, and BBB disruption^13^. Dysregulated molecular and ionic flux through a dysfunctional BBB would impact not only the NVU, but all cells in the brain^14^. The hippocampus, a key brain memory center^15,16^, is particularly affected, resulting in impaired neuronal connections and cognitive decline in vascular dementia^17–19^.

Obesity and dementia are characterized by common alterations in gene expression. Obesity can lead to gene expression changes in molecular pathways for amyloidogenesis, neuroinflammation, and apoptosis^20^. In human tissues, obesity and Alzheimer’s disease (AD) alter gene expression for pathogenic pathways for inflammation and mitochondrial dysfunction^21^. In rodents, expression of neuroinflammatory genes is increased in the hippocampus with obesity^22,23^ and AD^24^. In aged mice with high fat diet (HFD)-induced obesity, the hippocampal transcriptome is characterized by upregulated genes involved in microglial activation and negative regulation of synaptic plasticity, and downregulated genes involved in glial cell development and synaptic transmission^25^. However, these studies lack single cell resolution of gene expression analyses and only identified the effect of obesity for the average gene expression of tissues.

Single cell RNA (scRNA) sequencing technologies, including single nuclei RNA (snRNA) sequencing, identify transcriptomic alterations of different cell types and cell populations^26^. Thus, the National Institute on Aging prioritizes using these technologies to identify changes in the AD brain cell types during disease progression^27^. Presently, relatively few studies have used single cell technologies to investigate the impact of obesity on the brain, but others have investigated the impact of diet-induced obesity on various regions of the brain, including the hypothalamus^28,29^, midbrain^30^, and hippocampus^31,32^. Further, we recently used snRNA sequencing in the *ob/ob* mouse model of obesity to show that obesity induces cell-specific transcriptomic changes in hippocampal NVU cells of male mice, with implications for cognitive decline^33^.

Sex plays a key role in the development of obesity^34^; women have a higher prevalence of severe obesity than men^35^. Moreover, obesity-associated comorbidities including CVD, T2DM, dementia, and hypertension are more prevalent in women^36–38^, whereas hyperlipidemia and stroke affect men more often^39^. Additionally, there are sex differences in dementia; the prevalence of AD is higher in females^40^, while vascular dementia is more prominent in males^41^. According to the AD research network, there is mounting evidence that biologic sex is an important risk factor for the development and progression of dementia^42^. Sex differences in the brain transcriptome have been described in multiple brain regions, including the hippocampus^43–46^. Further, the effects of obesity on gene expression in the brain can differ between sexes. For example, HFD-induced obesity altered cortex gene expression in female mice, but had minimal effects in males^47^. Therefore, performing sex-based analyses^48,49^ that systematically examine the sex-based, biological determinants of health and disease, is critical to identify and understand sex-specific pathology and its impact on disease.

Although studies have been done in males, the effect of obesity on the hippocampal endothelial and NVU cell transcriptomes remains unexplored in females. Moreover, there is a lack of data on the sex differences in the molecular mechanisms of obesity-induced cerebrovascular dysfunction and its association with neurodegeneration. Data regarding cognitive function in obese female mice is also sparse. However, behavioral changes in both female and male *ob/ob* mice have been observed as early as 8 weeks of age^50^. Our own prior work, utilizing the current cohort of *ob/ob* mice, demonstrated behavior consistent with increased anxiety (a common symptom of dementia) on the open field test in female *ob/ob* mice at 15 weeks of age^51^. Thus, examining the transcriptomics of the female NVU and sex differences in the response to obesity will help provide a comprehensive view of the role that sex plays in the etiology of obesity induced dementia.

Therefore, in alignment with national research priorities, in this study we first determined the molecular mechanisms of obesity-induced transcriptomic alterations in the hippocampal NVU cell types (endothelial cells, microglial cells, astrocytes, and neurons) of female *ob/ob* mice, and its relevance to vascular dementia, using cutting-edge snRNA sequencing technology. Next, we assessed for sex-specific effects of obesity on the NVU using sex-based analyses^48,49^. By comparing to our prior study in males^33^ to identify important sex-specific and in common molecular mechanisms underlying obesity-associated cognitive impairment, we found that obesity modulates the transcriptome of the NVU in both a sex- and cell type-specific manner.

## Results

We confirmed the *ob/ob* phenotype in mice from the same cohort as the current study, demonstrating that both males and females are obese, hyperinsulinemic, hypercholesterolemic, and have impaired glucose tolerance as compared to wild-type (WT) mice^51^. As expected, there were also baseline sex differences in the NVU transcriptome between males and females, which differed by cell type (Supplementary Figs. 1 and 2, Supplementary Data 1–8). This stressed the importance of our study design comparing *ob/ob* mice to WT mice of the same sex in the analyses.

We therefore first analyzed transcriptomic changes in obese (*ob/ob*) females, as compared to WT females, for each NVU cell type. Subsequently, we performed sex-based analyses to characterize the impact of biologic sex on the effects of obesity in each of the cells of the NVU. The comparison utilized data we have previously published on male mice from the same cohort and with an identical study design to the current study^33^.

### Global transcriptomics uncovered cell type-specific expression profiles in females

**Fig. 1:**
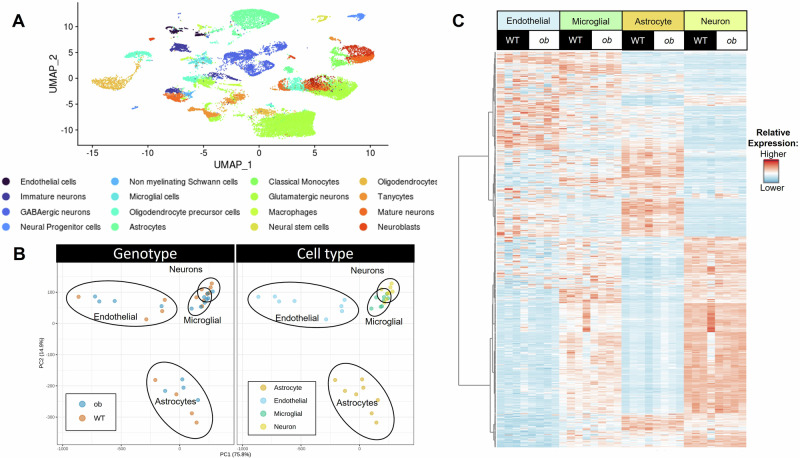
Effects of obesity on the global transcriptome of the NVU in female mice. **A** UMAP plot of sequenced nuclei from female *ob/ob* and WT mice. **B** PCA plot of the average expression values for each NVU cell type, color coded by genotype (*ob/ob* vs WT; left panel) and cell type (endothelial, microglial, astrocytes, neurons; right panel). Each dot represents one hippocampus. **C** Heatmap of the average expression values for each NVU cell type per sample. Shades of red indicate higher expression values while shades of blue represent lower expression values. Data shown are from *n* = 4 hippocampi per group. Average expression value source data can be found in Supplementary Data 10.

snRNA sequencing of female mouse hippocampal nuclei uncovered 16 cell subtypes (Fig. 1A). When examining cell type proportions, neurons were the most numerous, followed by astrocytes, in both *ob/ob* and WT female mice (Supplementary Data 9). There were no differences between female WT and *ob/ob* mice in the cell type composition of the hippocampal NVU nuclei sequenced. When examining all hippocampal cell types, the only difference was a lower proportion of non-myelinating Schwann cells in *ob/ob* mice. However, these cells were in very low abundance ( < 1% of sequenced cells) in both genotypes.

We restricted further analyses to cells of the NVU: endothelial cells, microglial cells, astrocytes, and neurons. When we assessed global gene expression (analyzing all genes rather than just those that were differentially expressed), separation was primarily determined by cell type, except for some co-clustering of microglial and neuronal cells (Fig. 1B). When all NVU cells were analyzed together, the global gene expression profile of *ob/ob* mice did not substantially differ from WT mice. Hierarchical clustering demonstrated higher expression levels for roughly half of the genes analyzed in microglial and neuronal cells, when compared to endothelial cells and astrocytes (Fig. 1C). Average expression values for each identified gene for NVU cells can be found in Supplementary Data 10.

### Obesity-induced differential gene expression is cell type-specific in the NVU of females

**Fig. 2:**
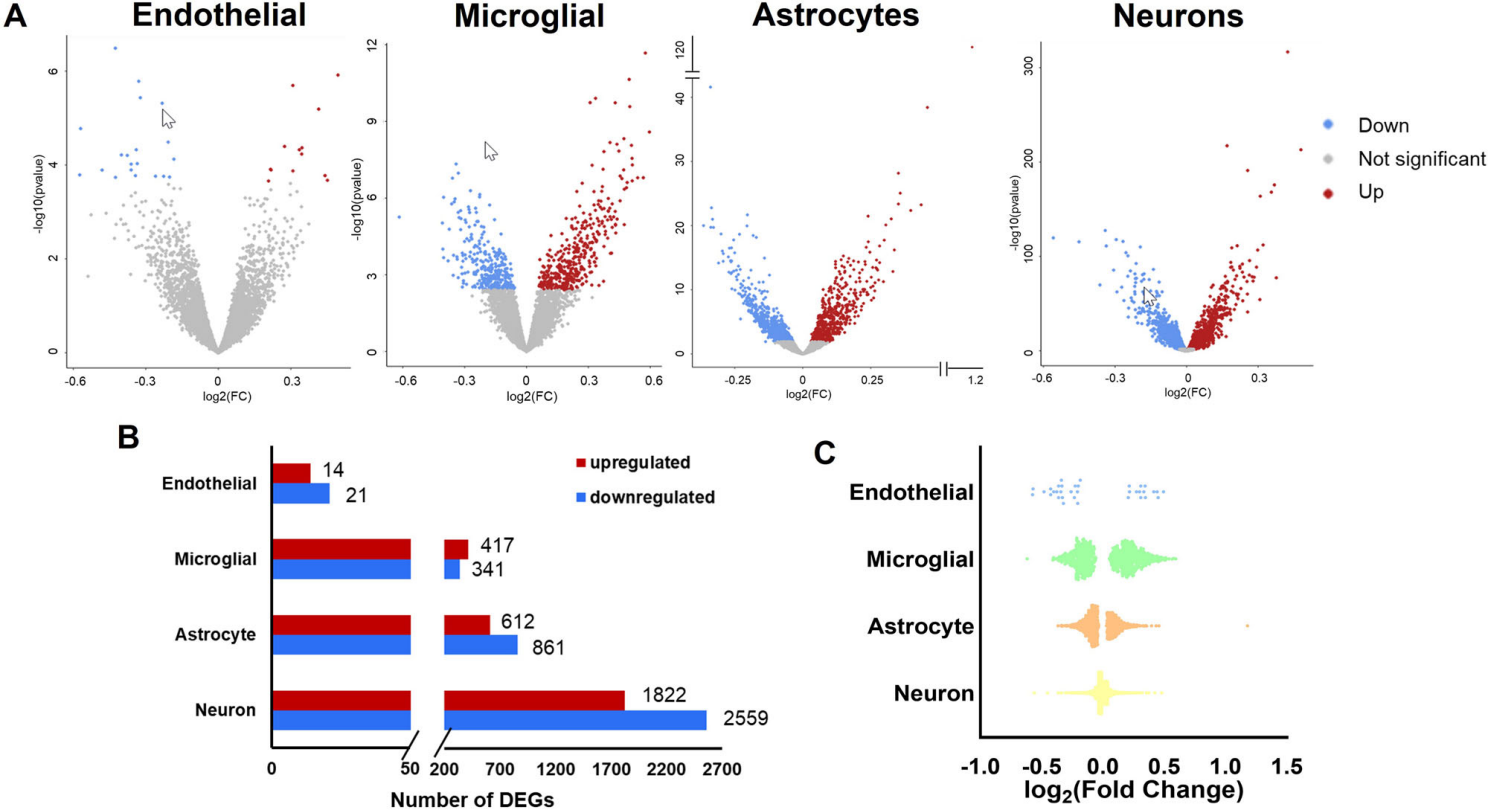
Obesity-induced DEGs in the hippocampal NVU cells of female mice. **A** Volcano plots of the DEGs for each NVU cell type. Each dot represents a gene; blue indicates downregulation and red indicates upregulation in *ob/ob* female mice as compared to WT female mice. Gray dots indicate genes which were not differentially expressed between *ob/ob* and WT female mice. **B** Bar chart of the number of upregulated (red bars) and downregulated (blue bars) DEGs in *ob/ob* female mice as compared to WT female mice. **C** Dot plot of the log2 fold change of the DEGs for each NVU cell type. Each dot represents a DEG. Data shown are from *n* = 4 hippocampi per group. Complete lists of DEGs for endothelial cells, microglial cells, astrocytes, and neurons can be found in Supplementary Data 11, 12, 13, and 14, respectively.

Next, we assessed differential expression induced by obesity for each NVU cell type individually, comparing female *ob/ob* mice to female WT mice. Obesity significantly altered the expression of genes in each of the four NVU cell types (Fig. 2A). Neurons had the most obesity-induced differentially expressed genes (DEGs), followed by astrocytes, microglial cells, and endothelial cells (Fig. 2B). The distribution of log_2_ fold changes in the obesity-induced DEGs of the NVU is provided in Fig. 2C. Complete lists of DEGs for endothelial cells, microglial cells, astrocytes, and neurons in females can be found in Supplementary Data 11, 12, 13, and 14, respectively. Our results indicated that obesity modulated differential gene expression in each of the cell types in the NVU of obese females, but the pattern of expression was cell-specific.

**Fig. 3:**
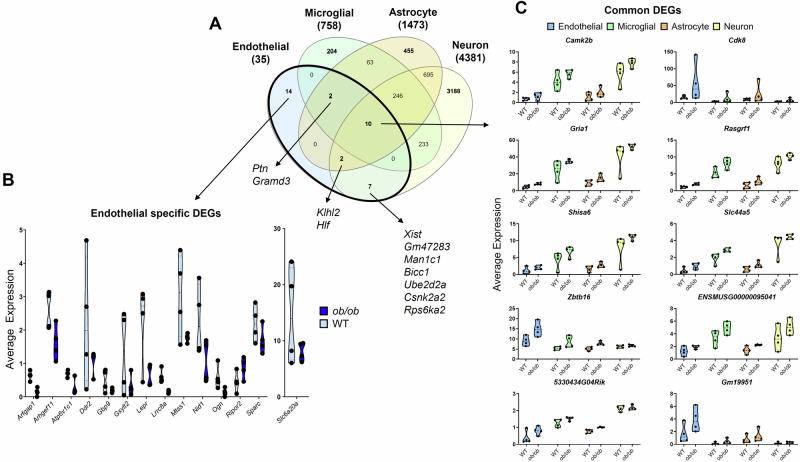
Comparison of obesity-induced DEGs among NVU cell types in females. **A** Venn diagram of DEGs in endothelial cells, microglial cells, astrocytes, and neurons. Selected DEGs specific to endothelial cells, and in common between endothelial cells and other NVU cell types, are shown. Violin plots of the endothelial-specific DEGs (**B**) and each of DEGs common to endothelial cells, microglial cells, astrocytes, and neurons (**C**). Black dots denote the average DEG expression for each sample (*n* = 4 hippocampi per group). Average expression value source data can be found in Supplementary Data 10. Complete lists of DEGs for endothelial cells, microglial cells, astrocytes, and neurons can be found in Supplementary Data 11, 12, 13, and 14, respectively.

We next compared obesity-induced DEGs in endothelial cells, as our primary area of interest, to those of the other NVU cell types. We identified that the proportions of cell-specific obesity-induced DEGs differed by cell type (Fig. 3A). In endothelial cells, microglial cells, and astrocytes cell-specific DEGs made up less than half of all DEGs, while in neurons cell-specific DEGs predominated. Obesity mostly downregulated endothelial-specific DEGs, including *Nid1*, *Sparc*, and *Ogn*, while *Ripor2* was the only upregulated endothelial-specific DEG (Fig. 3B). The majority of endothelial cell DEGs were shared with at least one other NVU cell type. Examples include: *Ptn-* downregulated by obesity in endothelial cells, microglial cells, and astrocytes; *Xist*- downregulated in both endothelial cells and neurons; and *Bicc1*- downregulated in endothelial cells and upregulated in neurons. Interestingly, the endothelial DEGs that were shared by all NVU cell types were upregulated, and included *Camk2b*, *Gria1*, *Shisa6, Slc44a5*, *and Zbtb16* (Fig. 3C).

### Functional analyses of DEGs revealed common pathways altered by obesity in the female NVU

**Fig. 4:**
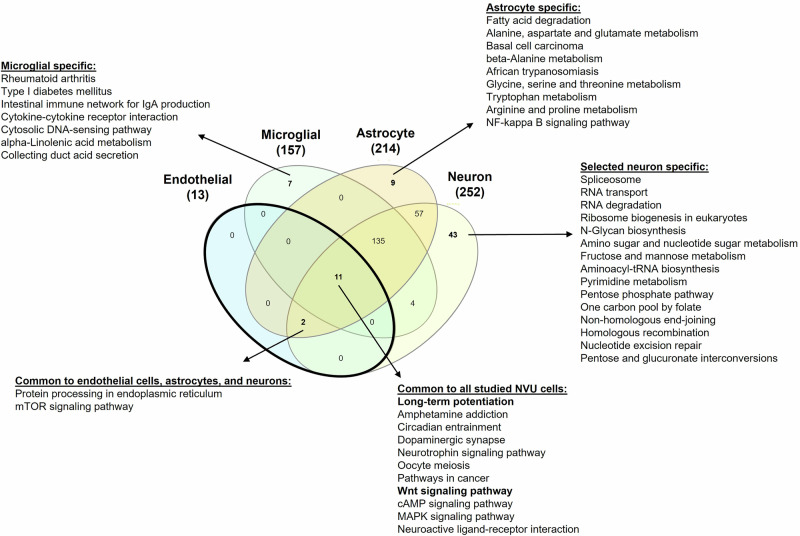
Comparison of overrepresented KEGG pathways altered by obesity among NVU cell types in female mice. A Venn diagram of the significantly overrepresented KEGG pathways based on the DEGs from each NVU cell type (endothelial cells, microglial cells, astrocytes, and neurons) is shown. Selected overrepresented pathways specific to NVU cell types and in common are listed. The bold circle emphasizes the comparisons in the context of endothelial cells. Complete lists of all significantly overrepresented pathways by obesity for endothelial cells, microglial cells, astrocytes, and neurons in females can be found in Supplementary Data 15, 16, 17, and 18, respectively.

We determined the overrepresented Kyoto Encyclopedia of Genes and Genomes (KEGG) pathways for the obesity-induced DEGs in each NVU cell type and then compared pathways amongst the cell types (Fig. 4). In endothelial cells, there were no cell-specific pathways; all overrepresented pathways were shared with at least one other NVU cell type. Endothelial cell pathways in common with astrocytes and neurons included mTOR signaling and protein processing in endoplasmic reticulum. Additionally, endothelial cell pathways shared with all other NVU cell types included long-term potentiation and Wnt signaling. There were cell-specific pathways in microglial cells (including cytokine-cytokine receptor interaction), astrocytes (including fatty acid degradation, NFκB signaling, and amino acid metabolism pathways), and neurons (including pathways related to the metabolism of nucleic acids and gene expression). Comprehensive lists of all significantly overrepresented pathways by obesity for endothelial cells, microglial cells, astrocytes, and neurons in females can be found in Supplementary Data 15, 16, 17, and 18, respectively. Thus, the pathways altered by obesity in endothelial cells were shared with other NVU cell types.

#### Correlation of gene expression to behavior in females

Our previous work demonstrated that female, but not male, *ob/ob* mice at 15 weeks of age exhibit increased anxiety behavior as evidenced by reduced time spent in the center of the open field test^51^. Thus, for each NVU cell type, we investigated whether the obesity-induced change in the expression levels of DEGs identified through snRNA sequencing in females correlated with the obesity-induced change in the percent time spent in the center of the open field (Supplementary Data 19). We found that in endothelial cells, the change in percent time in the center of the open field correlated with changes in expression for *Sparc* and *Gria1*. In microglial cells, 21 DEGs correlated with changes in open field performance, including *Oxct1* and *CD86*. Among the top 1000 most significant DEGs in astrocytes and neurons, 54 and 70 DEGs, respectively, significantly correlated with changes observed in open field behavior. They included *Adcy2* and *Smad 2* in astrocytes and *Mamld1* and *Sclt1* in neurons. Hence, our previously described obesity-induced behavioral changes^51^ correlated with the obesity-induced gene expression changes in the cells of the NVU.

### Sex as an important modifier of the global transcriptomic effects of obesity in the NVU

**Fig. 5:**
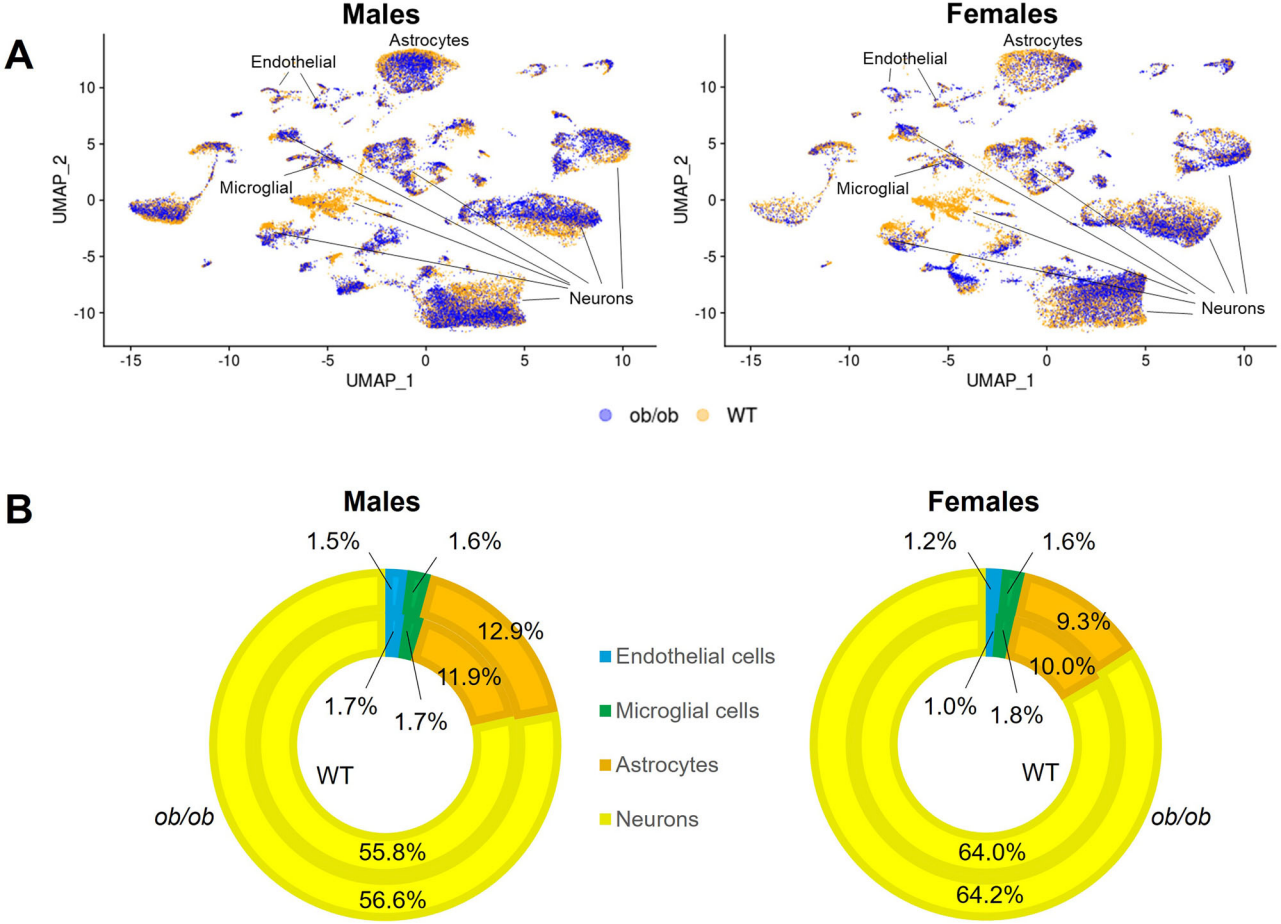
Effects of obesity on the cell type distribution of the hippocampus in males and females. **A** UMAP plots displaying the distribution of *ob/ob* (blue) and WT (yellow) nuclei in males and females. **B** Doughnut plots of the NVU cell type distribution in *ob/ob* (outer circle) and WT (inner circle) male and female mice. The percentages displayed indicate the average percent of sequenced nuclei. Data shown are from *n* = 4 hippocampi per group. Cell type composition source data are available in Supplementary Data 9.

To analyze sex differences, we first examined the global transcriptome using Uniform Manifold Approximation and Projection (UMAP). This revealed that male and female hippocampal cells formed similar overall cluster patterns. However, within these clusters, the distribution of *ob/ob* versus WT nuclei differed by sex (Fig. 5A). Next, we assessed the composition of the main NVU cell types—endothelial cells, microglial cells, astrocytes, and neurons—and found no genotype-based differences in either males or females (Fig. 5B). However, among *ob/ob* mice, females exhibited a higher proportion of neurons and a lower proportion of astrocytes compared to males. Comprehensive data on cell type composition and statistical analyses are available in Supplementary Data 9.

**Fig. 6:**
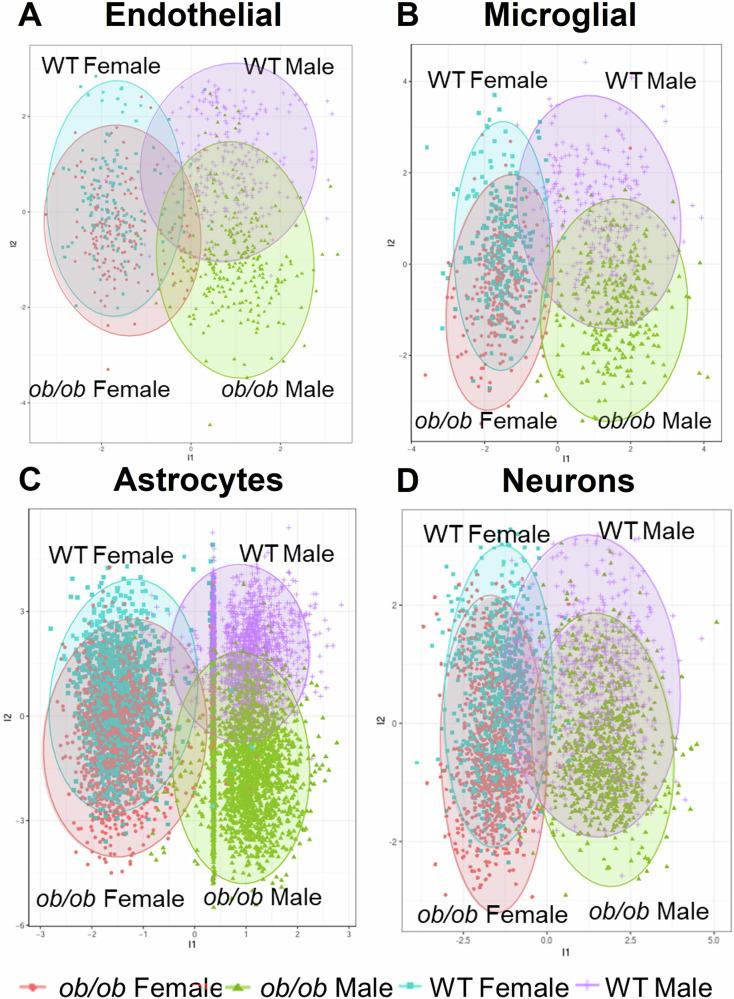
Comparison of the global transcriptomic profile of NVU cells in *ob/ob* and WT male and female mice. sPLS-DA plots of *ob/ob* and WT male and female mice in endothelial cells (**A**), microglial cells (**B**), astrocytes (**C**), and neurons (**D**). Each dot represents a nucleus derived from *n* = 4 hippocampi per group.

Next, we set out to understand how biological sex (male vs. female) influences the overall gene expression landscape in the main NVU cell types, comparing *ob/ob* mice to WT controls. Utilizing sparse partial least squares discriminant analysis (sPLS-DA), a statistical method designed to highlight the most informative genes that separate groups in high-dimensional data, we mapped each cell type’s global transcriptomic profile across sex and genotype. Our primary finding was that for each NVU cell type, the gene expression differences between males and females were more pronounced than those between *ob/ob* and WT (Fig. 6A–D). In other words, sex accounted for greater separation in their gene expression profiles than obesity did. For endothelial cells, microglial cells, and astrocytes, there was less overlap in *ob/ob* vs. WT profiles in males as compared to females (Fig. 6A–C). This indicates that obesity produced a stronger shift in gene expression in males than it did in females in those cell types. In contrast, for neurons (Fig. 6D), obesity produced similarly overlapping expression profiles in both sexes—meaning the effects of obesity on neurons were comparable in males and females. Thus, sex significantly alters how obesity affects gene expression, but the form of that modification varies by cell type within the NVU.

### Heterogeneity in the NVU cell DEG response to obesity between males and females

**Fig. 7:**
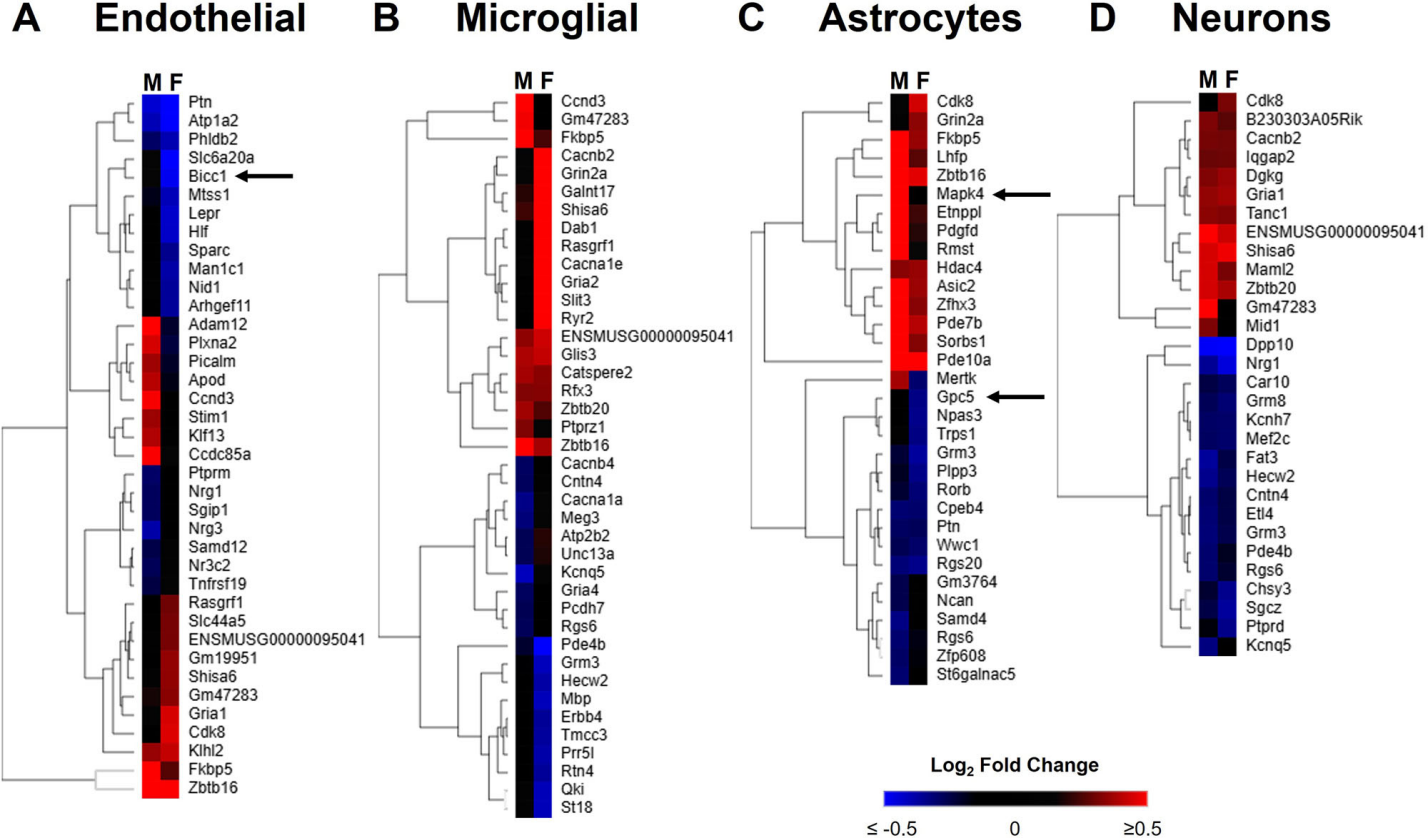
Cell type-specific patterns of heterogeneity in the response to obesity between males and females. The top 10 most upregulated and top 10 most downregulated DEGs induced by obesity (*ob/ob* vs. WT) for males (M) and females (F) were combined (with duplicates removed) into a heatmap for endothelial cells (**A**), microglial cells (**B**), astrocytes (**C**), and neurons (**D**). Euclidean hierarchical clustering was applied. In heatmaps, shades of red indicate a positive log_2_ fold change and shades of blue indicate a negative log_2_ fold change, black indicates a fold change at or close to zero, as indicated by the scale bar. Arrows indicate DEGs discussed in the text. Complete lists of female DEGs for endothelial cells, microglial cells, astrocytes, and neurons can be found in Supplementary Data 11, 12, 13, and 14, respectively. Complete lists of male DEGs for endothelial cells, microglial cells, astrocytes, and neurons can be found in Supplementary Data 20, 21, 22, and 23, respectively.

To compare how males and females respond to obesity, we generated heatmaps showing the log₂ fold change of the top obesity-induced DEGs in *ob/ob* versus WT mice. These heatmaps reveal distinct expression patterns in males and females. In endothelial cells (Fig. 7A) and microglial cells (Fig. 7B), most obesity-induced DEGs that appeared in females were not altered in males—and vice versa. For instance, *Bicc1* was strongly downregulated by obesity in female endothelial cells but remained unchanged in males. In astrocytes (Fig. 7C), males and females showed more similar DEG responses, though some genes—such as *Mapk4* (upregulated only in males) and *Gpc5* (downregulated only in females)—were sex-specific. In contrast, neurons (Fig. 7D) displayed largely overlapping obesity-induced DEGs between sexes. Overall, obesity-induced gene expression changes in the NVU varied by cell type, exhibiting heterogeneous, sex-dependent patterns.

**Fig. 8:**
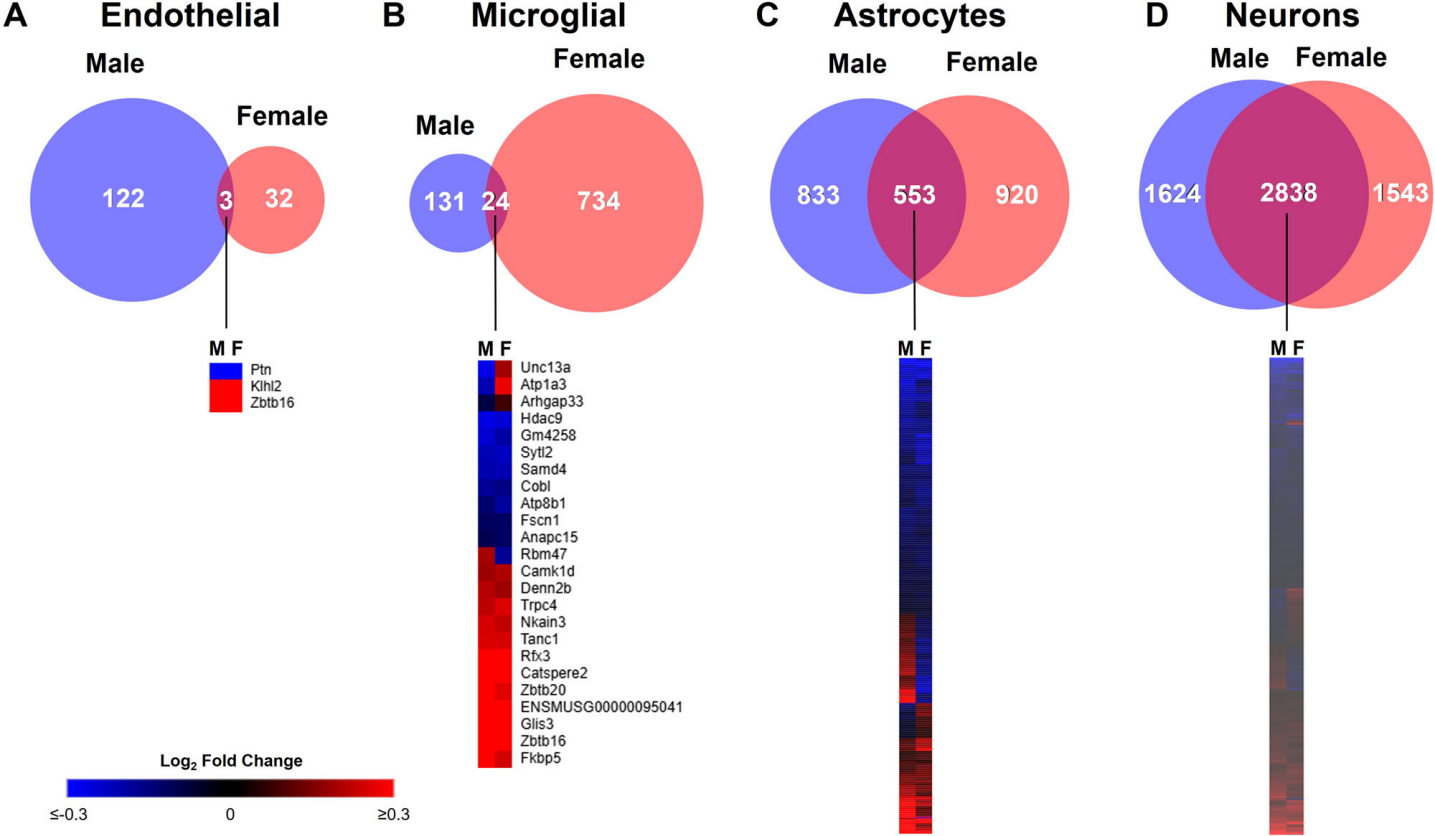
Comparison of male and female obesity-induced DEGS by cell type. Venn diagram of total number of DEGs in males and females, with corresponding heatmap of common DEGs, for endothelial cells (**A**), microglial cells (**B**), astrocytes (**C**), and neurons (**D**). In heatmaps, shades of red indicate a positive log_2_ fold change and shades of blue indicate a negative log_2_ fold change as indicated by the scale bar in males (M) and females (F). Complete lists of female DEGs for endothelial cells, microglial cells, astrocytes, and neurons can be found in Supplementary Data 11, 12, 13, and 14, respectively. Complete lists of male DEGs for endothelial cells, microglial cells, astrocytes, and neurons can be found in Supplementary Data 20, 21, 22, and 23, respectively.

Next, we compared all obesity-induced DEGs between males and females. This analysis revealed dramatic sex-specificity of DEGs as a hallmark of endothelial cells and microglial cells, with few common obesity-induced DEGs between males and females. In endothelial cells, there were nearly four times as many obesity-induced DEGs in males compared to females, with nearly all the DEGs being specific to either males or females (Fig. 8A). In microglial cells, we saw the opposite trend as females had nearly five times as many obesity-induced DEGs in females as compared to males and very few common DEGs between the sexes (Fig. 8B). However, four of the 24 common microglial DEGs were regulated in opposite directions by obesity in males and females (*Unc13a*, *Atp1a3*, and *Arhgap33* upregulated in females and downregulated in males; *Rbm47* upregulated in males and downregulated in females). In contrast, astrocytes (Fig. 8C) and neurons (Fig. 8D) shared many more obesity-induced DEGs between the sexes, although more than 20% of common DEGs were regulated in opposite directions by obesity in males and females. Complete lists of obesity-induced DEGs for endothelial cells, microglial cells, astrocytes, and neurons in males that were used in these comparisons can be found in Supplementary Data 20, 21, 22, and 23, respectively. Thus, sex affected which genes were differentially regulated by obesity, the cell type where the DEG regulation was most pronounced, and the pattern of differential expression.

### Sex-specificity predominated in overrepresented pathways of endothelial cells compared to other NVU cell types

**Fig. 9:**
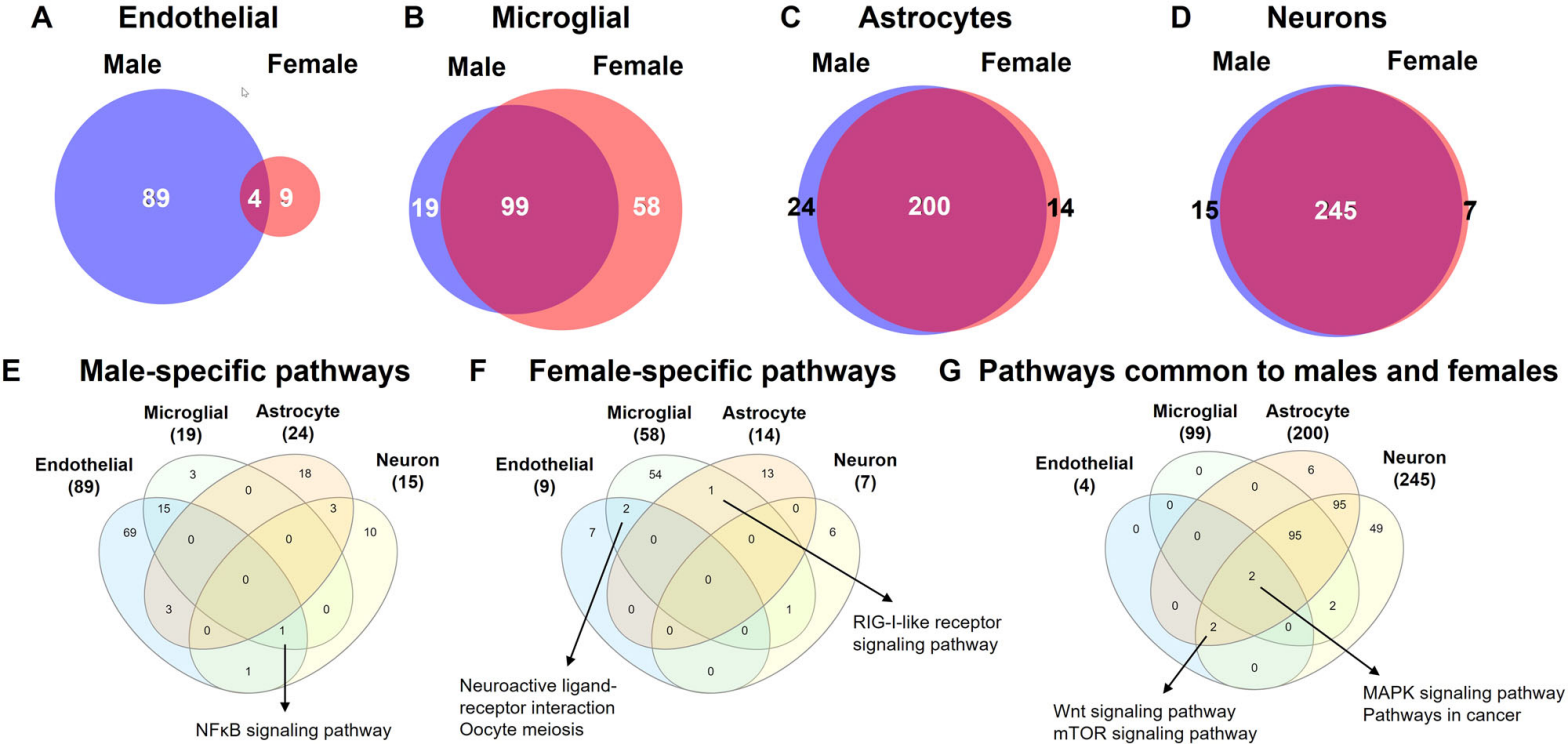
Comparison of overrepresented KEGG pathways altered by obesity between sexes, by cell type. Venn diagrams of overrepresented KEGG pathways modulated by obesity comparing males and females for endothelial cells (**A**), microglial cells (**B**), astrocytes (**C**), and neurons (**D**). Venn diagrams of male-specific (**E**), female-specific (**F**), and common (**G**) overrepresented KEGG pathways comparing the four NVU cell types. Selected overrepresented KEGG pathways are shown. Complete lists of all significantly overrepresented pathways in females for endothelial cells, microglial cells, astrocytes, and neurons can be found in Supplementary Data 15, 16, 17, and 18, respectively. Complete lists of all significantly overrepresented pathways in males for endothelial cells, microglial cells, astrocytes, and neurons can be found in Supplementary Data 24, 25, 26, and 27, respectively.

To further understand the impact of sex on the transcriptomic effects of obesity of the NVU, we compared obesity-altered overrepresented KEGG pathways between sexes (Fig. 9A–D). Comprehensive lists of all overrepresented pathways in males used in these comparisons for endothelial cells, microglial cells, astrocytes, and neurons can be found in Supplementary Data 24, 25, 26, and 27, respectively.

As with DEGs, endothelial cells displayed many more sex-specific overrepresented pathways than pathways in common between males and females (Fig. 9A). Male-specific pathways predominated and included insulin signaling, insulin resistance, focal adhesion, Pi3k-Akt signaling, Ras signaling, and Rap 1 signaling. Female-specific pathways included the cAMP signaling pathway. MAPK signaling, Wnt signaling, mTOR signaling, and pathways in cancer were the only overrepresented pathways common to males and females in endothelial cells.

In microglial cells, a greater proportion of pathways were common to males and females as compared to endothelial cells, yet over one third of female overrepresented pathways were female-specific (Fig. 9B), including Wnt signaling. Males did have some sex-specific pathways including the Jak-STAT signaling pathway.

In contrast, in astrocytes and neurons, the great majority of overrepresented pathways were common to males and females, with a small proportion of sex-specific pathways (Fig. 9C, D). The few sex-specific pathways identified in astrocytes included fatty acid biosynthesis (male-specific) and RIG-I-like receptor signaling pathway (female-specific). In neurons, pentose phosphate pathway (female-specific) and base excision repair (male-specific) were of the few sex-specific pathways identified.

Thus, as with DEGs, endothelial cells and microglial cells exhibited much more sex-specificity in obesity-altered pathways than astrocytes and neurons.

### Sex-specific pathways differed by cell type, while sex-independent pathways were shared by NVU cell types

When male-specific pathways were compared across the cell types, most pathways were found to be cell-specific (Fig. 9E). The greatest pathway commonalities were found between endothelial and microglial cells, with 15 pathways being common to these two cell types exclusively. Interestingly, NFκB signaling was a male-specific pathway for all cell types, except astrocytes (where it was female-specific).

Comparing female-specific pathways across the four NVU cell types demonstrated a primarily cell-specific pattern, with no pathways common to more than two NVU cell types (Fig. 9F). Examples of pathways shared between two cell types include, the neuroactive ligand-receptor interaction pathway (female-specific in both endothelial and microglial cells) and the RIG-I-like receptor signaling pathway (female-specific in both microglial cells and astrocytes).

Finally, we compared pathways common to both sexes across the cell types and discovered that these were pathways that were more likely to be shared across the cell types (Fig. 9G). For example, the mTOR and Wnt signaling pathways were shared between endothelial cells, astrocytes, and neurons. Furthermore, we found that the MAPK signaling pathway and pathways in cancer were common to both sexes and all four NVU cell types, likely representing a MAPK signaling mediated global effect of obesity on NVU function.

Our findings regarding cell type and sex effects of the NVU transcriptomic response to obesity are summarized below in the Discussion and conceptualized in Table 1.

**Table 1:** Conceptual summary of the impact of sex on the obesity-induced changes in the NVU transcriptome

Cell type	Sex-specificity	Effect size	Female-specific Pathways	Male-specific pathways	Shared pathways
Endothelial	+++	F«M	• 9 total: • Neurotrophin signaling • **cAMP signaling** • Long-term potentiation • Circadian entrainment • Dopaminergic synapse • Oocyte meiosis • Protein processing in endoplasmic reticulum • Neuroactive ligand-receptor interaction • Amphetamine addiction	• 89 total, including: • **NFκB signaling** • **PI3K-Akt signaling** • **Ras signaling** • **Rap1 signaling** • Jak-STAT signaling • **Insulin signaling** • **insulin resistance** • **Focal adhesion** • Cell adhesion molecules • Adherens junction	• 4 total: • **Wnt signaling** • **mTOR signaling** • **MAPK signaling** • Pathways in cancer
Microglial	++	F»M	• 58 total, including: • **Wnt signaling** • RIG-I-like receptor signaling • TGF-β signaling • Cell adhesion molecules • Tight junction • Adherens junction • Neuroactive ligand-receptor interaction • Oocyte meiosis • Glycerophospholipid metabolism • Glycerolipid metabolism • Ether lipid metabolism • Alpha-linolenic acid metabolism	• 19 total, including: • **NFκB signaling** • **Jak-STAT signaling** • Insulin signaling • VEGF signaling	• 99 total, including: • **MAPK signaling** • Pathways in cancer • cAMP signaling • Rap1 signaling • PI3K-Akt signaling • Long-term potentiation • Purine metabolism • Inositol phosphate metabolism
Astrocyte	+	F ~ M	• 14 total, including: • **RIG-I-like receptor signaling** • **NFkB signaling** • Glycolysis / gluconeogenesis • Galactose metabolism	• 24 total, including: • P53 signaling • Notch signaling • **Fatty acid biosynthesis** • Sphingolipid metabolism • Cholesterol metabolism	• 200 total, including: • **Wnt signaling** • **mTOR signaling** • **MAPK signaling** • Pathways in cancer • cAMP signaling • PI3K-Akt signaling • Insulin signaling • Insulin resistance • Neuroactive ligand-receptor interaction • Glycerophospholipid metabolism
Neuron	+	F ~ M	• 7 total: • Non-homologous end-joining • Terpenoid backbone biosynthesis • Pyrimidine metabolism • **Pentose phosphate pathway** • Pentose and glucuronate interconversions • Steroid biosynthesis • Legionellosis	• 15 total, including: • **NFκB signaling** • **Base excision repair** • Arginine and proline metabolism • beta-Alanine metabolism • Alanine, aspartate, and glutamate metabolism • Arginine biosynthesis	• 245 total, including: • **Wnt signaling** • **mTOR signaling** • **MAPK signaling** • Pathways in cancer cAMP signaling • PI3K-Akt signaling • Insulin signaling • Glutamatergic synapse • Serotonergic synapse • Dopaminergic synapse • Cholinergic synapse • GABAergic synapse • Insulin resistance

Key sex-specific or sex-independent pathways are highlighted with bold text.

## Discussion

In this study, we build on our recent work that examined the cell-specific effects of obesity in the hippocampal NVU in male mice utilizing snRNA sequencing^33^. We first studied the effects of obesity on the female hippocampal NVU transcriptome and its relationship to the behavioral changes we previously reported^51^. We subsequently performed sex-based analyses to elucidate the similarities and differences between sexes in the response of the NVU to obesity with relevance to cognitive dysfunction. We discuss our findings in the context of (1) the NVU response to obesity in females with a focus on endothelium, given our interest in the vascular contributions to dementia, (2) how the transcriptomic changes correlate to changes in cognitive behavior in females, (3) sex as a modifier of the response to obesity, and (4) sex-independent responses.

Endothelial cells in females exhibited obesity-induced DEGs, both endothelial-specific and shared with other NVU cell types. The endothelial-specific obesity-induced DEGs downregulated by obesity included *Nid1*, *Sparc, and Ogn*, which play a role in angiogenesis^52–55^. Moreover, *Sparc* also regulates transendothelial permeability and endothelial barrier function^54,56^. Among the shared DEGs, *Ptn*, (downregulated by obesity in endothelial cells, microglial cells, and astrocytes), plays a role in angiogenesis, mitogenesis, and differentiation^57^ and is reduced in the brains of persons with AD^58^. *Xist* (downregulated in endothelial cells and neurons), a lncRNA with a well-known role in X chromosome inactivation^59^, and *Bicc1* (downregulated in endothelial cells and upregulated in neurons) have both been shown to promote angiogenesis^60,61^. Taken together, DEGs downregulated in endothelial cells by obesity have implications for impaired angiogenesis and alterations in BBB permeability, functional impairments seen in dementia.

Within the DEGs shared by endothelial cells and all other NVU cell types in females, two functional themes emerged, neurotransmission and autophagy. Three of the common DEGs altered by obesity in all NVU cell types (*Slc44a5*, *Gria1*, and *Shisa6)* play a key role in neurotransmission. *Slc44a5* encodes choline transporter-like protein 5, which transports choline, a molecule important for neurotransmission^62^, across the plasma membrane^63^. *Gria1* encodes an excitatory synaptic transmission receptor, and is an important gene in AD^64^. *Shisa6* encodes a protein that interacts with AMPA-type glutamate receptors, which mediate fast excitatory synaptic transmission^65^. Furthermore, two of the common DEGs, *Camk2b and Zbtb16*, have a role in autophagy^66–68^, which, when dysregulated, can contribute to neurodegeneration in diseases such as AD^69^.

There were no endothelial-specific overrepresented pathways in response to obesity in females, but endothelial cells did share overrepresented pathways with other NVU cell types. Long-term potentiation and Wnt signaling were common to all four studied NVU cell types. Long-term potentiation in the hippocampus is important in memory^70^. The Wnt proteins preserve brain homeostasis by regulating BBB stability, synaptic plasticity, and inflammation^71,72^. Additionally, anormal Wnt signaling occurs in aging and has been described in AD and in vascular brain injury^72,73^. Furthermore, mTOR signaling was common between endothelial cells, astrocytes, and neurons. mTOR signaling is inhibitory of autophagy, plays a crucial role in cellular proliferation and migration (functions important in cellular repair), contributes to cerebrovascular dysfunction, and plays a role in many neurodegenerative diseases, including dementia^74–78^. Thus, altered neurotransmission and autophagy may be key mechanisms in the NVU contributing to the increased risk of dementia from obesity in females.

We have previously shown that *ob/ob* female mice exhibited decreased time spent in the center of an open field compared to WT female mice^51^, indicating that obesity increases anxiety behavior. Correlation analyses revealed a significant positive correlation between the change in the percent time spent in the center of the open field and the change in expression of DEGs including: *Sparc* in endothelial cells, *Oxct1* in microglia, *Adcy2* in astrocytes, and *Mamld1* in neurons. Prior studies have shown that these genes are dysregulated in neurodegenerative diseases such as AD^79,80^, Huntington disease^81^, and Parkinson’s disease^82^. This suggests that the changes in gene expression modulated by obesity in the NVU of females correlate with maladapted cognitive behavior, indicating that obesity may play a role in neurodegeneration.

Our data demonstrated that sex was an important modifier of the NVU transcriptomic response to obesity, but its impact differed by cell type. In endothelial cells, over 90% of obesity-induced DEGs were sex-specific. Further, there was an overall greater number of DEGs and overrepresented pathways that obesity modulated in males compared to females. Some of the male-specific overrepresented pathways in endothelial cells included insulin signaling and insulin resistance, pathways of consequence as insulin resistance associated hyperinsulinemia promotes impaired cognition^83^. Focal adhesion, PI3K-Akt, Ras, and Rap1 signaling were additional male-specific overrepresented pathways in endothelial cells. They play a key role in endothelial cell survival, proliferation, and BBB disruption in neurodegenerative diseases^14,84,85^. In contrast to males, there were fewer obesity-altered pathways unique to females, which included the cAMP signaling pathway. The cAMP signaling pathway regulates endothelial tight junctions and maintains the BBB integrity^86^. Thus, our studies revealed that obesity alters pathways connected to the BBB in endothelial cells in a sex-specific manner.

In a trend opposite to endothelial cells, in microglial cells females exhibited nearly five times as many obesity-induced DEGs as males. Although the Wnt signaling pathway was common to both sexes in all other NVU cell types, it was female-specific in microglia. The Wnt signaling pathway regulates microglial inflammation associated with AD pathogenesis^87^. One of the few male-specific pathways in microglia was the Jak-STAT signaling pathway, which promotes neuroinflammation in neurodegeneration^88^. Thus, in microglial cells, obesity altered inflammation through different pathways in males and females, yet the affected pathways had implications for cognitive decline in both sexes.

In astrocytes and neurons, most DEGs were shared by males and females. However, the direction of change was opposite in males and females (upregulated in one sex but down regulated in the other) for more than 20% of the shared DEGs. Similarly, most overrepresented pathways in astrocytes and neurons were shared between males and females. However, we identified a few sex-specific pathways modulated by obesity in astrocytes and neurons. In astrocytes, fatty acid biosynthesis was a male-specific pathway. Interestingly, we have previously shown increased brain fatty acyls in obese mice, with a greater impact in males^51^, and others have shown dysregulation of fatty acid metabolism in AD pathogenesis^89^. On the other hand, the RIG-I-like receptor signaling pathway was female-specific in astrocytes. RIG-I-like receptor signaling plays a role in the innate immune response of astrocytes and has been implicated in the progression of AD^90,91^. In neurons, the base excision repair pathway was overrepresented only in males. Impairment of base excisions repair (a key DNA repair pathway) is associated with AD^92^. In contrast, the pentose phosphate pathway was altered by obesity in females only. Impairment of the pentose phosphate pathway has been implicated in the progression of AD^93^. Therefore, although sex had a lesser impact on the effect of obesity in astrocytes and neurons as compared to endothelial cells and microglia, astrocytes and neurons did exhibit sex differences in the response to obesity, with relevance to dementia.

NFκB signaling was a male-specific pathway altered by obesity in neurons, endothelial cells, and microglia, whereas it was female-specific in astrocytes. NFκB signaling is well known for its proinflammatory function and plays a neurotoxic role in AD^94^. Our finding is supported by others that have noted sex differences in NFκB signaling in a mouse AD model^95^. Thus, the NFκB signaling pathway appears to be a key sex-specific pathway throughout the NVU.

Our findings identified only two overrepresented pathways that were independent of sex in all four NVU cell types, pathways in cancer and the MAPK signaling pathway. The MAPK (mitogen-activated protein kinase) signaling pathways include c-Jun N-terminal kinase, p38 MAPK, and extracellular signal-regulated kinase. They are involved in a wide range of cellular processes including apoptosis and inflammation, and have been implicated in neurodegenerative diseases, including AD^96^. Thus, this well studied pathway is a potential mechanism by which obesity affects the NVU in both males and females, and therefore, is likely a primary conserved pathway contributing to increased dementia risk from obesity in both sexes.

Additional common overrepresented pathways, in endothelial cells, astrocytes, and neurons, in both males and females were mTOR and Wnt signaling pathways. Interestingly, Wnt signaling was common to all NVU cell types in females, being female-specific in microglial cells. These signaling pathways are discussed above and are involved in autophagy and neurotransmission. Indeed, others have also implicated disruption of mTOR and Wnt signaling in obesity and neurogenerative diseases^72,97,98^. Thus, the effects of obesity in the hippocampal NVU that are independent of sex and shared by most NVU cells pertain to key functions relevant to cognitive decline, including autophagy and neurotransmission.

A key strength of our study was that snRNA sequencing allowed us to focus on cell type specific transcriptomics and examine the NVU specifically, whereas bulk RNA sequencing restricts analyses to data averaged across a tissue^99^. We also addressed sex differences by thoroughly analyzing females (an understudied group), then performing sex-based analyses, thereby delving into the historically overlooked effect of biologic sex. This approach permitted us to derive many unique previously unreported findings. However, our work does have limitations. For instance, snRNA sequencing does not provide data on the location of gene expression within the hippocampus. Follow-up studies could use other methodologies, such as spatial transcriptomics and in situ hybridization, to validate and determine the location of differential expression of key genes observed in obesity. Additionally, not all changes in gene expression translate to protein expression changes; future studies could examine this with immunofluorescence and confocal microscopy. Furthermore, future research should examine sex differences in BBB permeability and measure microglial activation and neuroinflammation in response to obesity (which were beyond the scope of our studies) since DEGs in endothelial cells and microglia in the present study were associated with pathways linked to these functional outcomes. Future research should also assess the progression of transcriptomic changes at other timepoints in the mouse lifespan, such as with aging models.

In conclusion, our study uncovered the obesity-induced gene expression changes in the female NVU at a cellular level and compared to that of males, providing deeper insight into the mechanisms of obesity-linked cognitive dysfunction.

Our data established that in females:

1. obesity modulated differential gene expression changes in the NVU in a cell type-specific manner, which correlated with maladapted cognitive behavior.
2. obesity altered endothelial specific DEGs which were mostly downregulated and involved in angiogenesis and BBB permeability.
3. the majority of endothelial obesity-induced DEGs, and all overrepresented pathways, were shared with other NVU cells, including those related to neurotransmission and autophagy.

Our subsequent sex-based analyses revealed that the impact of obesity on the NVU transcriptome was altered by sex in a cell type-specific manner (for our conceptual summary see Table 1). We demonstrated that:

1. there was significant sex-specificity in the obesity-induced DEGs and overrepresented pathways in endothelial cells and microglia, while in astrocytes and neurons there was much more commonality between sexes.
2. NFκB signaling was a sex-specific pathway for all NVU cell types (male-specific in endothelial cells, microglial cells, and neurons; female-specific in neurons).
3. MAPK signaling was a key sex-independent process in all NVU cell types, and the Wnt and mTOR signaling pathways were common to males and females in all NVU cell types, except microglial cells. These pathways may present an important mechanistic opportunity for identifying treatment targets applicable to both sexes.

The findings from our work provide a new outlook on cell- and sex-specificity in the transcriptomic response of the hippocampal neurovascular unit to obesity, whereby sex-specific and sex-independent approaches to prevention and treatment strategies for dementia in obesity may need to be considered.

## Methods

### Experimental animals

We have complied with all relevant ethical regulations for animal use. Research was conducted in conformity with the Public Health Service Policy on Humane Care and Use of Laboratory Animals and reported in accordance with the ARRIVE guidelines. The institutional review board of the University of California, Davis, the Institutional Animal Care and Use Committee, approved a protocol detailing the research question, all procedures, and planned analyses which was prepared prior to the study (protocol number 22598, initial approval: December 14, 2021, subsequent reviews: December 01, 2022 and November 30,2023). Food and water intake, as well as activity, was monitored daily by vivarium staff to ensure the wellbeing of the animals. All procedures were conducted to reduce any discomfort to the mice. Our prespecified humane endpoints were: the persistent inability to reach food or water for more than 12 h; a 20% decrease in body weight; a reduced body condition score; or the development of conditions that result in significant pain that cannot be alleviated by analgesics. No adverse events were encountered in this study. The experimental unit for this study was one mouse. The criteria for inclusion in the study was that the mice survived until the terminal timepoint for tissue collection. For all animals the procedures described below were carried out in the same order and at the same timepoints relative to mouse age, to minimize confounders. The primary outcome measure of our study was to determine the transcriptomic response to obesity in female mice utilizing snRNA sequencing. Our secondary outcome measure was to compare the response to obesity in females and males (from our previously published data^33^).

Female *ob/ob* mice (B6.Cg-Lep<ob > /J strain and 000632 stock) and female wild-type (WT) control mice (C57Bl/6 J strain and 000664 stock) were received from Jackson Laboratories (Bar Harbor, ME, USA) at 10 weeks of age. Mice were housed individually in duplex cages in a temperature- and humidity-controlled environment with a 12-h light/dark cycle at the University of California, Davis Mouse Biology Program. Throughout the study, mice were fed the standard AIN-93M purified diet (catalog number, TD.00102 Envigo Teklad diets, Madison, WI, USA) comprised of 68.3% carbohydrate, 12.4% protein, and 4.1% fat (w/w) *ad libitum*. Mice were housed under these conditions until tissue collection at 18 weeks of age. We selected the *ob/ob* model for this study due to the fact that it is one of the most common and well characterized mouse model of obesity^100,101^. Additionally, the *ob/ob* model exhibits an obese phenotype similar to humans with comorbidities of impaired glucose tolerance, hyperinsulinemia, and hyperlipidemia^100,102,103^, making it an appropriate model of human obesity.

At 18 weeks of age, mice were fasted for 8 h, then euthanized by exsanguination under anesthesia (216–270 mg/kg ketamine / 24–30 mg/kg xylazine), to ensure no pain. Immediately after exsanguination the brain tissue was removed (from *n* = 4 mice per genotype, total of 8 mice). One hemisphere of hippocampal region was quickly dissected and immediately frozen in the vapor phase of liquid nitrogen. Blinding was not possible during tissue collection due to obvious phenotype differences. Hippocampi were stored at –80 °C until nuclei isolation. We chose to collect brain tissue for snRNA sequencing at 18 weeks of age based on reports that male *ob/ob* mice exhibit memory deficits between 13–23 weeks of age^104–107^. An experimental timeline, including previously published analyses performed on mice from the same cohort indicated relative to the endpoints reported in the current study, can be found in Supplementary Fig. 3.

### Hippocampal nuclei isolation and fixation

snRNA sequencing technology has advantages over scRNA sequencing when it comes to reduced dissociation bias, compatibility with frozen samples, and elimination of dissociation-induced transcriptional stress responses^108^, hence we chose to use snRNA sequencing for our work. Nuclei isolation and fixation was done as previously described, keeping the samples on ice or in a refrigerated centrifuge throughout the process^109^. Briefly, the frozen hippocampal tissue was homogenized in a Dounce homogenizer (Catalog # K8853000001, DWK Life Sciences Kimble™ Kontes™ Dounce Tissue Grinders). The homogenate was filtered using a 40 µm cell strainer and the nuclei were pelleted by centrifugation (600 × *g* for 4 min). Nuclei were washed once with PBS supplemented with RNAse inhibitors and 0.0075% bovine serum albumin and pelleted by centrifugation (600 g for 4 min). The nuclei were resuspended in PBS supplemented with RNAse inhibitors and filtered through a 40 µm cell strainer. The nuclei concentration was then determined using a hemocytometer. Up to 3,000,000 nuclei per isolated hippocampus (*n* = 4 per group, 8 total) were fixed according to the manufacturer’s instructions, with centrifugation speed adjusted to 300 × *g*, using the Nuclei Fixation Kit (Catalog # SB1003, Parse Biosciences, Seattle, WA). The number of replicates was chosen to balance cost and precision^110^. The fixed nuclei in suspension was stored at –80 °C, as per the manufacturer’s guidelines, until library preparation for snRNA sequencing.

### Library preparation and sequencing

The library preparation and sequencing was performed by the UC Davis DNA Technologies and Expression Analysis Core. The core personnel were blinded to the group allocation of the samples they received. Barcoded single nuclei libraries were prepared from fixed hippocampal single nuclei suspensions using the Parse Evercode single nuclei technology (Evercode Whole Transcriptome Mega kit, Catalog # EC-W01050, Parse Biosciences, Seattle, WA) according to manufacturer’s instructions. The cDNA and library fragment size distribution were verified on a Bioanalyzer 2100 (Agilent) and TapeStation (Agilent), respectively. The results indicated that there was minimal RNA degradation, and it was appropriate to proceed with the snRNA sequencing process. The libraries were quantified by fluorometry on a Qubit instrument (LifeTechnologies) and by qPCR with a Kapa Library Quant kit (Kapa Biosystems-Roche) prior to sequencing. We sequenced two sublibraries on a NovaSeq 6000 sequencer (Illumina) with paired-end 100 bp reads, which provided sequences for approximately 3500 nuclei for each sample. The sequencing generated approximately 35,000 reads per nuclei. Read quality was assessed by Q30 in the barcodes. Sequencing characteristics, alignment metrics and library characteristics can be found in Supplementary Data 28.

### Bioinformatic analyses of obesity effects in female mice

To assess for baseline sex differences, we compared WT male mice (male data previously published^33^; GSE262249) to WT female mice. To address the transcriptomic changes induced by obesity in the NVU in female mice we compared female *ob/ob* to female WT mice. Randomization was not used to assign mice to groups as group assignment was based on genotype. Processing of the snRNA sequencing data was performed with assistance of the UC Davis Bioinformatics core. Raw sequencing data were preprocessed and combined using Parse Biosciences’ split-pipe pipeline (v0.9.6p). Expression matrices were imported into Seurat^111^ for downstream analyses including: RNA quality assessment, merging of data, normalization, and clustering.

The quality of snRNA sequencing data was assessed using the nFeature_RNA plot (number of detected genes per nuclei), the nCount_RNA plot (the number of detected Unique Molecular Identifiers (UMIs) in each nuclei), and the percent.mito plot (the percentage of mitochondrial genes per nuclei). These data can be found in Supplementary Fig. 4. No experimental units were excluded from analyses; for nuclei, filtering was conducted to retain nuclei that had 500–10,000 genes expressed, 1000–50,000 UMIs detected, and <5% mitochondrial reads. After filtering, the nuclei classified as doublet using DoubletFinder^112^ were removed. The remaining data for all samples was merged, normalized using “LogNormalize” mode, and scaled to regress out cell cycle effect and sequencing depth (using the number of UMI as a proxy).

The first 50 principal components clustered the nuclei using the “FindCluster” function with shared nearest neighbor modularity optimization based clustering algorithm^113^ at resolution level 2 to generate UMAP embeddings. Cell types were identified using R package ScType^114^ with brain and immune system markers. From the identified cell types in the entire hippocampus, we chose to focus our differential expression analyses on the cells of the NVU, endothelial cells, microglial cells, astrocytes, and neurons. Cells referred to as neurons in our analyses were a combination of the following neuronal sub-types: “GABAergic neurons”, “glutamatergic neurons”, “immature neurons”, “interneurons”, “mature neurons” and “neurons”.

Differentially expressed genes (DEGs) were determined by comparing *ob/ob* female mice to WT female mice. ggplot2 R package^115^ was used to create visualizations, specifically to display the log2 fold changes and the number of DEGs (defined as genes with an FDR adjusted *p* value < 0.06). The FDR value cut off of 0.06 was selected to be consistent with the previously published male data which we are comparing to^33^. Additionally, others have used an adjusted *p* value as high as *p* < 0.1 as a cutoff for identifying DEGs in RNA sequencing studies, thus a cutoff of <0.06 is not outside the range normally used in RNA sequencing studies^116,117^. No fold change cutoff was applied. GeneTrai2l^118^ was utilized to identify overrepresented KEGG pathways of DEGs using the Benjamini and Hochberg False Discovery Rate (FDR) adjusted *p* value < 0.05.

### Sex-based analyses

To determine the sex-specific and sex independent effects of obesity on the NVU, we performed sex-based analyses^48,49^, incorporating sex (male/female) as a biologic variable into our research design, disaggregating data by sex in the collection, analysis, and data interpretation, as well as identifying intersectional relationships, utilizing the female data from this study and our previously published data on an identical study on *ob/ob* males^33^. Global profiles were compared by sPLS-DA. All bioinformatic analyses were conducted in the same manner in males and females, including determination of DEGs and pathway analyses, to ensure that the only variable being studied was the effect of sex.

### Statistics and reproducibility

The analyses pertained to the single nuclei sequencing results of 4 hippocampi per group (from 4 individual mice per group: female *ob/ob* vs female WT). To determine whether there were differences in the cell type composition we utilized a two-sided Student’s *t* test when the assumptions that data was normally distributed (as determined by Shapiro-Wilk test) and equal variances (as determined by the F test) were met. If the assumption of equal variances was not met a *t* test with Welch’s correction as used. In the case that the assumption of normal distribution was not met, the Mann-Whitney test was used to compare groups.

The PCA of global gene expression data for females was generated using ExpressAnalyst^119^ and the heatmap was generated using MetaboAnalyst^120^. Volcano plots of the DEGs were generated using Galaxy platform^121^. InteractiVenn^122^ and BioVenn^123^ were used to produce Venn diagrams. s-PLSDA plots of nuclei for the comparison of male and female response to obesity were generated by the mixOmics R package^124^. For neurons, due to the large number of nuclei, only a subset of the nuclei are shown in the sPLS-DA. The combined neurons except for “interneurons” were randomly selected for 2985 nuclei, then the selected 2985 nuclei were combined with the interneuron nuclei (all were used due to very low number) for the sPLS-DA. The number of components used for the model is 15. Definitions of the methodologies and visualization tools used can be found in the Supplementary Definitions.

To identify potential links between *ob/ob* and WT differences in the expression of genes and the behavioral effects of obesity, we assessed whether the difference in expression of DEGs correlated with the difference in the percent time spent in the center of the open field test, data which was previously published^51^. Pearson’s correlation coefficients were determined for the difference between *ob/ob* and the average WT percent time spent in the center of the open field and all differentially expressed genes identified in endothelial cells and microglia, as well as the 1,000 most significant DEGs (as determined by lowest *p* value) in astrocytes and neurons were determined using SRplot^125^.

### Reporting summary

Further information on research design is available in the Nature Portfolio Reporting Summary linked to this article.

## Supplementary information

Supplementary Information

Description of Additional Supplementary Files

Supplementary Data 1-28

Reporting Summary

## Data Availability

The snRNA sequencing data used in this manuscript is available in the NCBI GEO database under accession numbers GSE263863 (female data) and GSE262249 (male data). Source data for all graphs and charts can be found within the sequencing data available at the NCBI GEO database or in Supplementary Data 1–28. All other data are available from the corresponding authors on reasonable request.
