# Supplementary material for: Cell- and sex-specificity in the transcriptomic response of the hippocampal neurovascular unit to obesity
Source: Commun Biol. 2025 Nov 27;8:1712. doi: 10.1038/s42003-025-09112-6 (PMC12660818; doi:10.1038/s42003-025-09112-6)
Supplement: Supplementary file 1 — Supplementary Information [file 42003_2025_9112_MOESM1_ESM.pdf]

Cell- and Sex-Specificity in the Transcriptomic Response of the Hippocampal Neurovascular Unit to Obesity
Supplementary Information

Jennifer E. Norman[1]*, Saivageethi Nuthikattu[1], Dragan Milenkovic[2,3], Amparo C. Villablanca[1]*

[1]Division of Cardiovascular Medicine, Department of Internal Medicine, University of California, Davis, Davis, CA, USA
[2]Department of Nutrition, University of California, Davis, Davis, CA, USA
[3]Plants for Human Health Institute, Food Bioprocessing and Nutrition Sciences Department, North Carolina State University, Kannapolis, NC, USA
*Corresponding authors

Supplementary Figures

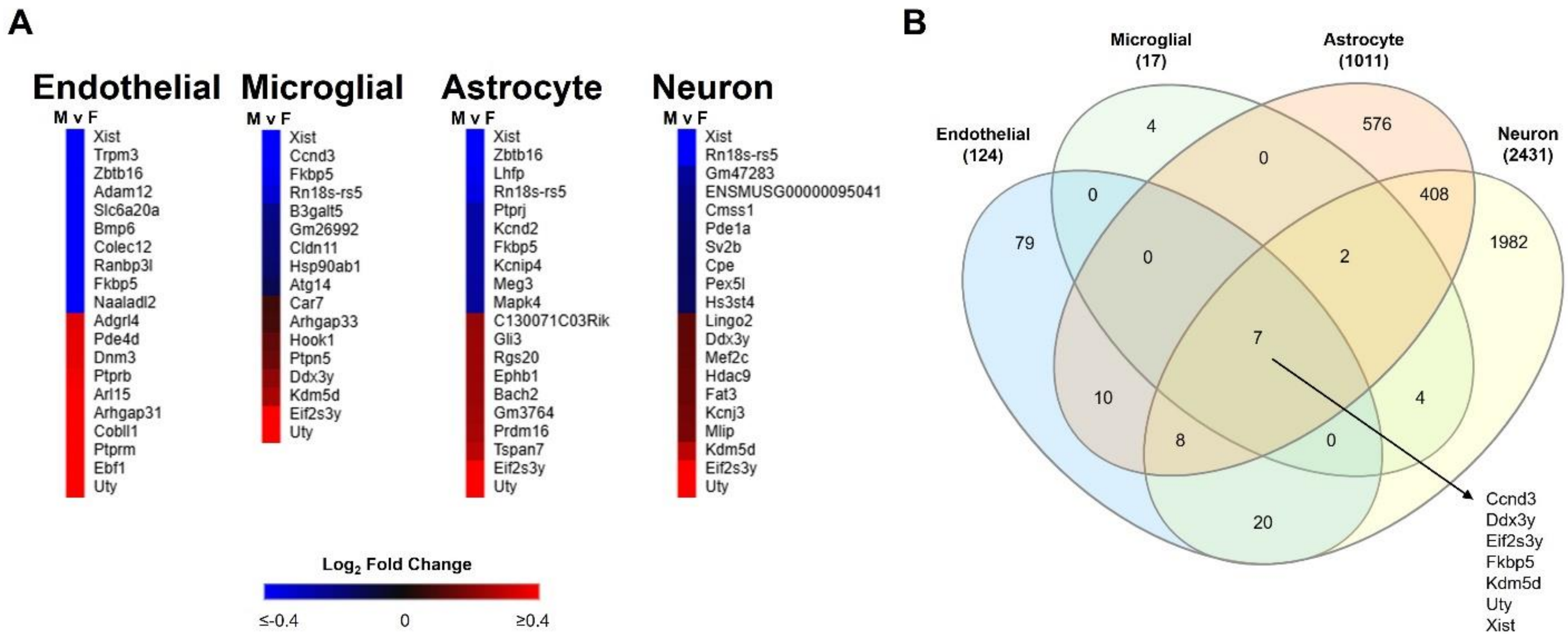

Supplementary Figure 1. Comparison of male vs female DEGs for WT mice between cell types. A) Heatmaps of the $\log_2$ fold change of the top 10 upregulated and top 10 downregulated DEGs in male (M) as compared to female (F) WT hippocampal NVU cells (endothelial cells, microglial cells, astrocytes, and neurons). Red indicates an upregulated gene, while blue indicates a downregulated gene in males as compared to females, as indicated by the scale bar. B) Venn diagram comparing all male vs female DEGs between NVU cell types. Complete lists of DEGs between male and female WT hippocampal endothelial cells, microglial cells, astrocytes, and neurons can be found in Supplementary Data 1, 2, 3, and 4, respectively.

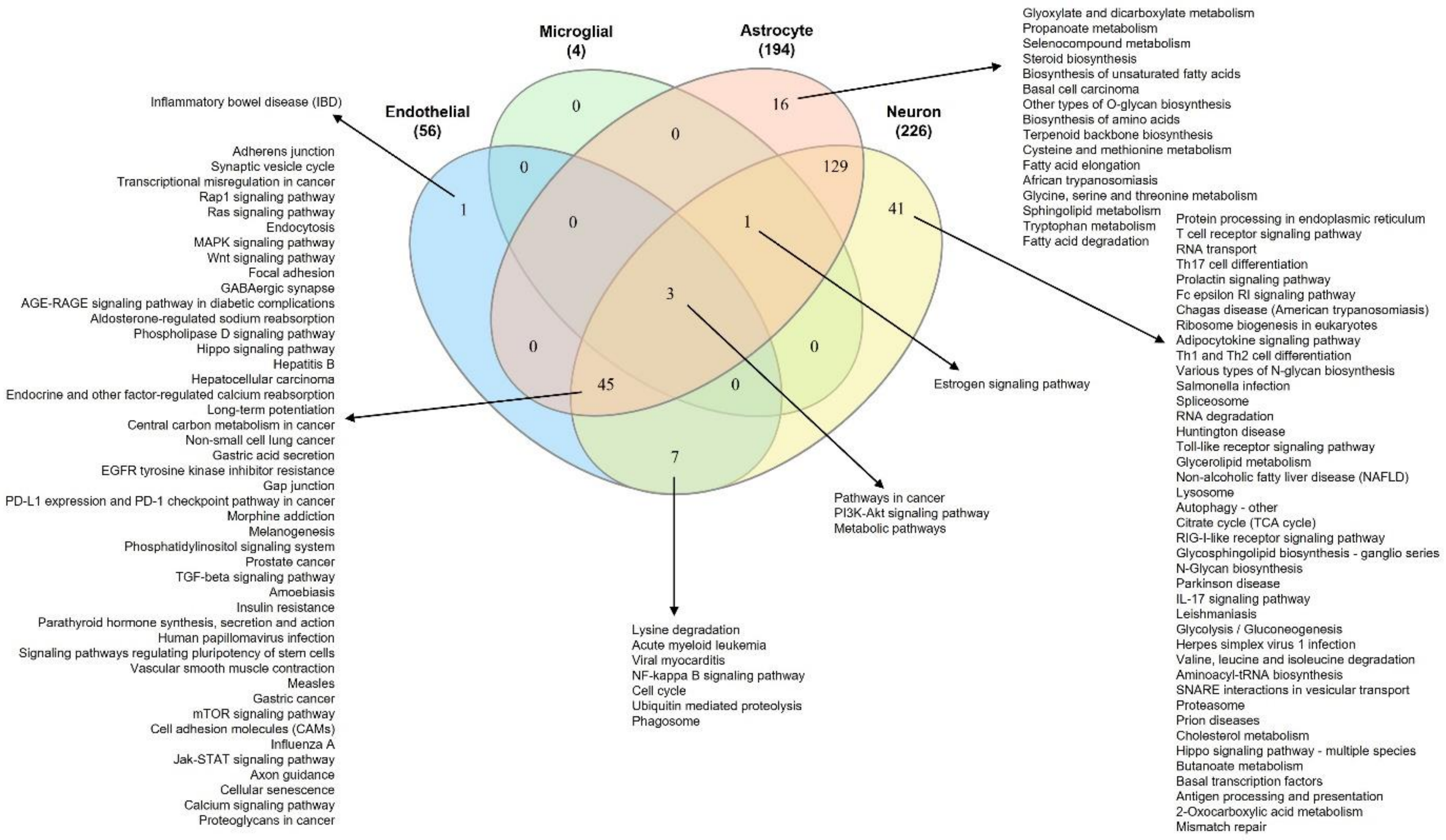

Supplementary Figure 2. Comparison of overrepresented KEGG pathways differing between males and females in WT mice among NVU cell types. A Venn diagram of the significantly overrepresented KEGG pathways based on the WT male vs female DEGs from each NVU cell type (endothelial cells, microglial cells, astrocytes, and neurons) is shown. Selected pathways are listed. Complete lists of overrepresented KEGG pathways for endothelial cells, microglial cells, astrocytes, and neurons can be found in Supplementary Data 5, 6, 7, and 8, respectively.

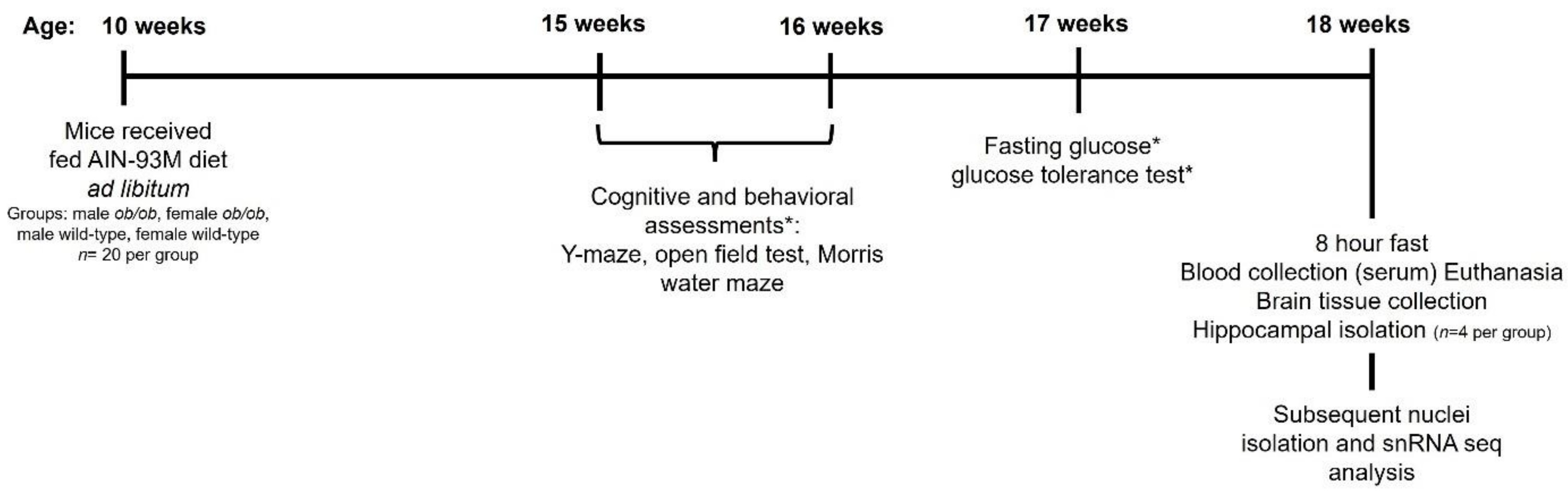

*cognitive and behavioral assessments as well as fasting serum values and glucose tolerance test were previously published in Norman et al. 2024
DOI: 10.3390/ijms25063475

Supplementary Figure 3. Experimental timeline.

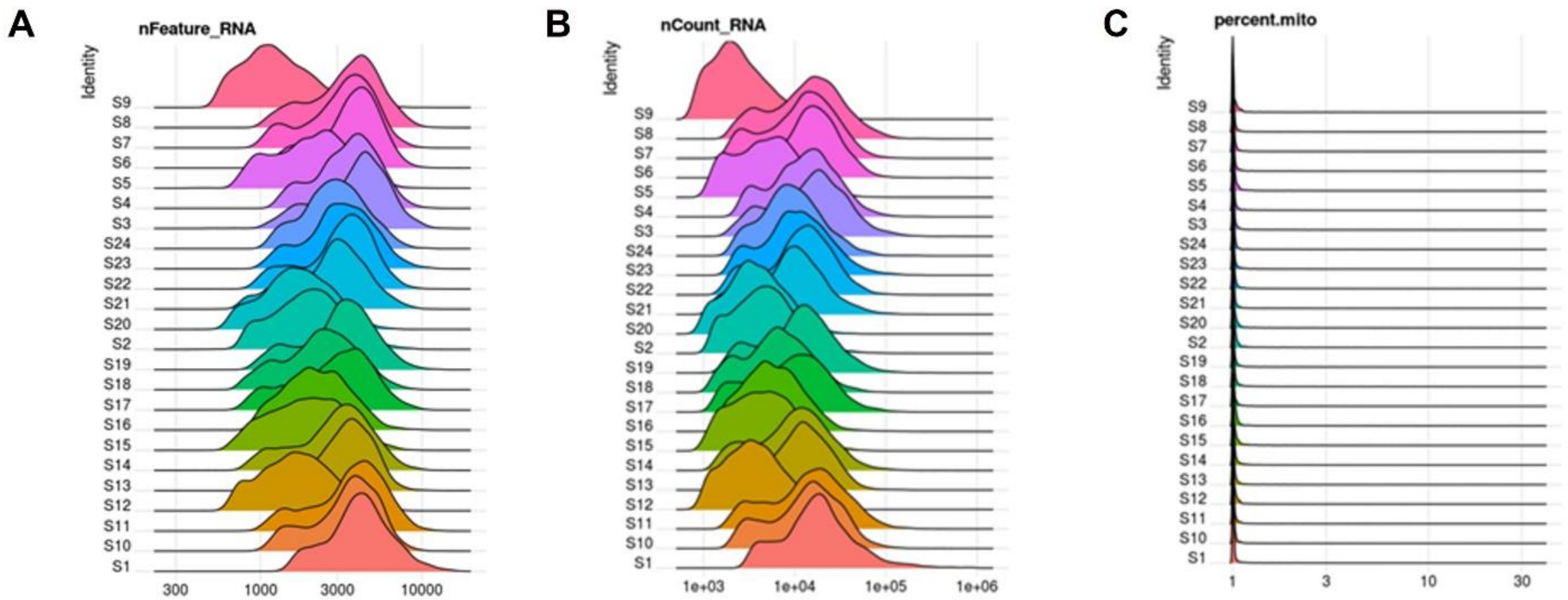

Supplementary Figure 4. snRNAseq data quality assessment plots.
A) The nFeature_RNA plot shows the number of detected genes in each nucleus. B) The nCount_RNA plot shows the number of detected Unique Molecular Identifiers (UMIs) in every nucleus. C) The percent.mito plot indicates the percentage of mitochondrial genes in each nucleus. The female *ob/ob* samples in this study were S15, S16, S20, and S23 and WT samples in this study were S6, S12, S18, and S24. The male *ob/ob* samples in this study were S13, S14, S19, and S22 and WT samples in this study were S5, S11, S17, and S21. The other samples shown were sequenced at the same time but are from other studies and not included in the analyses for this paper.

Supplementary Definitions

- **Uniform Manifold Approximation and Projection (UMAP)** in snRNA-seq analysis reduces high-dimensional gene expression data to 2D or 3D and hence preserving data structure. This helps in visualizing nuclei clusters, identifying distinct cell types, and interpreting complex relationships, and therefore enhancing the understanding of cellular diversity and interactions.

- **Principal component analysis (PCA)** is a tool to visualize distance and relatedness between populations, the results of which are usually discussed in terms of component scores for each population studied.

- **Heatmaps** represent gene expression levels uses color gradients to depict the abundance of gene expression across samples. Darker or more intense colors often indicate higher expression levels, while lighter colors represent lower expression. This visualization helps identify patterns and differences in gene expression across various conditions or samples.

- **Volcano plots** visually represent the magnitude and significance of differentially expressed genes.

- **Sparse Partial Least Squares Discriminant Analysis (sPLS-DA)** is a supervised method to discriminate sample groups. It builds on Partial Least Squares (PLS), a statistical technique that models relationships between predictors and responses by identifying orthogonal components that maximize covariance between these variables. PLS is particularly effective for high-dimensional or collinear data, reducing dimensionality while retaining essential information. sPLS-DA combines PLS with variable selection to focus on the most relevant features, enhancing classification accuracy and interpretability. This approach identifies key biomarkers, aiding in the classification of nuclei and revealing cell type differences in complex snRNA seq datasets.

- **False Discovery Rate (FDR)** is a statistical method used to correct for multiple comparisons in hypothesis testing. It adjusts p-values to control the expected proportion of false positives among the significant results. FDR helps reduce the likelihood of incorrectly identifying results as statistically significant due to chance.
