# Supplementary material for: Cell- and sex-specificity in the transcriptomic response of the hippocampal neurovascular unit to obesity
Source: Commun Biol. 2025 Nov 27;8:1712. doi: 10.1038/s42003-025-09112-6 (PMC12660818; doi:10.1038/s42003-025-09112-6)
Supplement: Supplementary file 2 — Description of Additional Supplementary Files [file 42003_2025_9112_MOESM2_ESM.docx]

**Description of Additional Supplementary files**

File name: Supplementary Data 1

Description: Complete list of endothelial DEGs in male WT mice as compared to female WT mice.

File name: Supplementary Data 2

Description: Complete list of microglial DEGs in male WT mice as compared to female WT mice.

File name: Supplementary Data 3

Description: Complete list of astrocyte DEGs in male WT mice as compared to female WT mice.

File name: Supplementary Data 4

Description: Complete list of neuron DEGs in male WT mice as compared to female WT mice.

File name: Supplementary Data 5

Description: Complete list of overrepresented KEGG pathways differing between males and females in endothelial cells of WT mice.

File name: Supplementary Data 6

Description: Complete list of overrepresented KEGG pathways differing between males and females in microglial cells of WT mice.

File name: Supplementary Data 7

Description: Complete list of overrepresented KEGG pathways differing between males and females in astrocytes of WT mice.

File name: Supplementary Data 8

Description: Complete list of overrepresented KEGG pathways differing between males and females in neurons of WT mice.

File name: Supplementary Data 9

Description: Cell type percent composition of sequenced nuclei.

File name: Supplementary Data 10

Description: Average expression for each NVU cell type in each sample from *ob/ob* and WT female mice.

File name: Supplementary Data 11

Description: Complete list of endothelial DEGs in female *ob/ob* mice as compared to female WT mice.

File name: Supplementary Data 12

Description: Complete list of microglial DEGs in female *ob/ob* mice as compared to female WT mice.

File name: Supplementary Data 13

Description: Complete list of astrocyte DEGs in female *ob/ob* mice as compared to female WT mice.

File name: Supplementary Data 14

Description: Complete list of neuron DEGs in female *ob/ob* mice as compared to female WT mice.

File name: Supplementary Data 15

Description: Complete list of overrepresented KEGG pathways modulated by obesity in endothelial cells in female mice.

File name: Supplementary Data 16

Description: Complete list of overrepresented KEGG pathways modulated by obesity in microglial cells in female mice.

File name: Supplementary Data 17

Description: Complete list of overrepresented KEGG pathways modulated by obesity in astrocytes in female mice.

File name: Supplementary Data 18

Description: Complete list of overrepresented KEGG pathways modulated by obesity in neurons in female mice.

File name: Supplementary Data 19

Description: Significant correlations between changes in gene expression and changes in the percent time in the center of the open field in *ob/ob* female mice as compared to WT female mice.

File name: Supplementary Data 20

Description: Complete list of endothelial DEGs in male *ob/ob* mice as compared to male WT mice.

File name: Supplementary Data 21

Description: Supplementary Data 21. Complete list of microglial DEGs in male *ob/ob* mice as compared to male WT mice.

File name: Supplementary Data 22

Description: Complete list of astrocyte DEGs in male *ob/ob* mice as compared to male WT mice.

File name: Supplementary Data 23

Description: Complete list of neuron DEGs in male *ob/ob* mice as compared to male WT mice.

File name: Supplementary Data 24

Description: Complete list of overrepresented KEGG pathways modulated by obesity in endothelial cells in male mice.

File name: Supplementary Data 25

Description: Complete list of overrepresented KEGG pathways modulated by obesity in microglial cells in male mice.

File name: Supplementary Data 26

Description: Complete list of overrepresented KEGG pathways modulated by obesity in astrocytes in male mice.

File name: Supplementary Data 27

Description: Complete list of overrepresented KEGG pathways modulated by obesity in neurons in male mice.

File name: Supplementary Data 28

Description: snRNA sequencing characteristics, alignment metrics, and library characteristics for samples from female WT and *ob/ob* mice.
