# Supplementary material for: Cell- and sex-specificity in the transcriptomic response of the hippocampal neurovascular unit to obesity
Source: Commun Biol. 2025 Nov 27;8:1712. doi: 10.1038/s42003-025-09112-6 (PMC12660818; doi:10.1038/s42003-025-09112-6)
Supplement: Supplementary file 4 — Reporting Summary [file 42003_2025_9112_MOESM4_ESM.pdf]

nature portfolio

Corresponding author(s): Amparo C. Villablanca
Jennifer E. Norman

Last updated by author(s): Oct 10, 2025

Reporting Summary

Nature Portfolio wishes to improve the reproducibility of the work that we publish. This form provides structure for consistency and transparency in reporting. For further information on Nature Portfolio policies, see our Editorial Policies and the Editorial Policy Checklist.

Statistics

For all statistical analyses, confirm that the following items are present in the figure legend, table legend, main text, or Methods section.

n/a	Confirmed	
☐	☒	The exact sample size (*n*) for each experimental group/condition, given as a discrete number and unit of measurement
☐	☒	A statement on whether measurements were taken from distinct samples or whether the same sample was measured repeatedly
☐	☒	The statistical test(s) used AND whether they are one- or two-sided<br>*Only common tests should be described solely by name; describe more complex techniques in the Methods section.*
☒	☐	A description of all covariates tested
☐	☒	A description of any assumptions or corrections, such as tests of normality and adjustment for multiple comparisons
☐	☒	A full description of the statistical parameters including central tendency (e.g. means) or other basic estimates (e.g. regression coefficient) AND variation (e.g. standard deviation) or associated estimates of uncertainty (e.g. confidence intervals)
☐	☒	For null hypothesis testing, the test statistic (e.g. *F*, *t*, *r*) with confidence intervals, effect sizes, degrees of freedom and *P* value noted<br>*Give P values as exact values whenever suitable.*
☒	☐	For Bayesian analysis, information on the choice of priors and Markov chain Monte Carlo settings
☒	☐	For hierarchical and complex designs, identification of the appropriate level for tests and full reporting of outcomes
☐	☒	Estimates of effect sizes (e.g. Cohen's *d*, Pearson's *r*), indicating how they were calculated

Our web collection on statistics for biologists contains articles on many of the points above.

Software and code

Policy information about availability of computer code

Data collection	No software was used for data collection.
Data analysis	Raw sequencing data were preprocessed and combined using Parse Biosciences' split-pipe pipeline (v0.9.6p). Expression matrices were imported into Seurat for downstream analyses. Cell types were identified using R package ScType. ggplot2 R package was used to create visualizations. GeneTrail was utilized to identify overrepresented KEGG pathways of DEGs. The PCA of global gene expression data for females was generated using ExpressAnalyst and the heatmap was generated using MetaboAnalyst. Volcano plots of the DEGs were generated using Galaxy platform. InteractiVenn and BioVenn were used to produce Venn diagrams. s-PLSDA plots of nuclei for the comparison of male and female response to obesity were generated by the mixOmics R package. SRplot was used to determine Pearson's correlation coefficients. Graphpad Prism was used to conduct t tests and Mann Whitney tests.

For manuscripts utilizing custom algorithms or software that are central to the research but not yet described in published literature, software must be made available to editors and reviewers. We strongly encourage code deposition in a community repository (e.g. GitHub). See the Nature Portfolio guidelines for submitting code & software for further information.

Data

Policy information about availability of data

All manuscripts must include a data availability statement. This statement should provide the following information, where applicable:

- Accession codes, unique identifiers, or web links for publicly available datasets
- A description of any restrictions on data availability
- For clinical datasets or third party data, please ensure that the statement adheres to our policy

The snRNA sequencing data used in this manuscript is available in the NCBI GEO database under accession numbers GSE263863 (female data) and GSE262249 (male data). Source data for all graphs and charts can be found within the sequencing data available at the NCBI GEO database and in Supplementary Data 1-28.

Research involving human participants, their data, or biological material

Policy information about studies with human participants or human data. See also policy information about sex, gender (identity/presentation), and sexual orientation and race, ethnicity and racism.

Reporting on sex and gender	No human participants.
Reporting on race, ethnicity, or other socially relevant groupings	No human participants.
Population characteristics	No human participants.
Recruitment	No human participants.
Ethics oversight	No human participants.

Note that full information on the approval of the study protocol must also be provided in the manuscript.

Field-specific reporting

Please select the one below that is the best fit for your research. If you are not sure, read the appropriate sections before making your selection.

☒ Life sciences ☐ Behavioural & social sciences ☐ Ecological, evolutionary & environmental sciences

For a reference copy of the document with all sections, see nature.com/documents/nr-reporting-summary-flat.pdf

Life sciences study design

All studies must disclose on these points even when the disclosure is negative.

Sample size	n=4 hippocampi (from 4 individual mice) was chosen as a sample size, based on a balance between cost and precision. No sample size calculation was performed, however we based our sample size on our own prior snRNA sequencing work and that published by others.
Data exclusions	No data was excluded.
Replication	DEGs were determined based on statistical significants with 4 biological replicates. We have not yet repeated this study, due to the costly nature of snRNA sequencing.
Randomization	Randomization was not possible in our study as the group assignment was based on the genotype of the mice (ob/ob or WT).
Blinding	Blinding was not possible during tissue collection due to the obvious phenotypic differences between ob/ob and WT mice. However, the core facility that performed the barcoding, library preparation, and sequencing analyses were blinded as to which genotype of mouse the samples they processed came from.

Reporting for specific materials, systems and methods

We require information from authors about some types of materials, experimental systems and methods used in many studies. Here, indicate whether each material, system or method listed is relevant to your study. If you are not sure if a list item applies to your research, read the appropriate section before selecting a response.

Materials & experimental systems

n/a	Involved in the study	
☒	☐	Antibodies
☒	☐	Eukaryotic cell lines
☒	☐	Palaeontology and archaeology
☐	☒	Animals and other organisms
☒	☐	Clinical data
☒	☐	Dual use research of concern
☒	☐	Plants

Methods

n/a	Involved in the study	
☒	☐	ChIP-seq
☒	☐	Flow cytometry
☒	☐	MRI-based neuroimaging

Animals and other research organisms

Policy information about studies involving animals; ARRIVE guidelines recommended for reporting animal research, and Sex and Gender in Research

Laboratory animals	Female ob/ob mice (B6.Cg-Lep<ob>/J strain and 000632 stock) and female wild-type (WT) control mice (C57Bl/6J strain and 000664 stock) were received from Jackson Laboratories (Bar Harbor, ME, USA) at 10 weeks of age. They were housed in our facilities until collection of tissues at 18 weeks of age.
Wild animals	This study did not involve wild animals.
Reporting on sex	Sex was a primary consideration in our study as we first addressed the effect of obesity in female mice specifically, then compared to our previous work in males to assess sex differences. Throughout our study data was disaggregated by sex and we addressed sex as a biological variable.
Field-collected samples	This study did not involve samples collected from the field.
Ethics oversight	The Institutional Animal Care and Use Committee at UC Davis, approved a protocol detailing the research question, all procedures, and planned analyses which was prepared prior to the study (protocol number 22598, initial approval: December 14, 2021, subsequent reviews: December 01, 2022 and November 30,2023).

Note that full information on the approval of the study protocol must also be provided in the manuscript.

Plants

Seed stocks	No plants were used in this study.
Novel plant genotypes	No plants were used in this study.
Authentication	No plants were used in this study.
